# Neurofilaments can differentiate ALS subgroups and ALS from common diagnostic mimics

**DOI:** 10.1038/s41598-021-01499-6

**Published:** 2021-11-11

**Authors:** Arvin Behzadi, Fani Pujol-Calderón, Anton E. Tjust, Anna Wuolikainen, Kina Höglund, Karin Forsberg, Erik Portelius, Kaj Blennow, Henrik Zetterberg, Peter Munch Andersen

**Affiliations:** 1 Department of Clinical Sciences, Neurosciences, Umeå University, 901 85 Umeå, Sweden; 2 Department of Psychiatry and Neurochemistry, University of Gothenburg, Gothenburg, Sweden; 3 Department of Chemistry, Umeå University, Umeå, Sweden; 4 Clinical Neurochemistry Laboratory, Sahlgrenska University Hospital, Mölndal, Sweden; 5 Department of Neurodegenerative Disease, UCL Institute of Neurology, London, UK; 6 UK Dementia Research Institute at UCL, London, UK

**Keywords:** Neurological disorders, Motor neuron disease, Amyotrophic lateral sclerosis, Diagnostic markers, Medical research, Molecular medicine, Neurology

## Abstract

Delayed diagnosis and misdiagnosis are frequent in people with amyotrophic lateral sclerosis (ALS), the most common form of motor neuron disease (MND). Neurofilament light chain (NFL) and phosphorylated neurofilament heavy chain (pNFH) are elevated in ALS patients. We retrospectively quantified cerebrospinal fluid (CSF) NFL, CSF pNFH and plasma NFL in stored samples that were collected at the diagnostic work-up of ALS patients (n = 234), ALS mimics (n = 44) and controls (n = 9). We assessed the diagnostic performance, prognostication value and relationship to the site of onset and genotype. CSF NFL, CSF pNFH and plasma NFL levels were significantly increased in ALS patients compared to patients with neuropathies & myelopathies, patients with myopathies and controls. Furthermore, CSF pNFH and plasma NFL levels were significantly higher in ALS patients than in patients with other MNDs. Bulbar onset ALS patients had significantly higher plasma NFL levels than spinal onset ALS patients. ALS patients with *C9orf72HRE* mutations had significantly higher plasma NFL levels than patients with *SOD1* mutations. Survival was negatively correlated with all three biomarkers. Receiver operating characteristics showed the highest area under the curve for CSF pNFH for differentiating ALS from ALS mimics and for plasma NFL for estimating ALS short and long survival. All three biomarkers have diagnostic value in differentiating ALS from clinically relevant ALS mimics. Plasma NFL levels can be used to differentiate between clinical and genetic ALS subgroups.

## Introduction

Amyotrophic lateral sclerosis (ALS) is an adult onset fatal neurodegenerative syndrome characterized by the insidious onset of progressive motor symptoms and signs secondary to the loss of upper and lower motor neurons and their tracts^1^. In the majority of patients, symptoms start in a limb, termed spinal onset ALS, or in the head and neck region, named bulbar onset ALS^1^. The median diagnostic delay is approximately a year^2^ which is a long time for a condition with a median survival time from onset of paresis of only 30 months^3^. ALS is heterogeneous, misdiagnoses are frequent, and it is a challenge in clinical practice to determine an ALS diagnosis^4^. It is essential in the diagnostic process to exclude a number of conditions termed ALS mimics that present with symptoms similar to ALS and may be difficult to differentiate^5–7^. Approximately 10% of ALS patients self-report a family history of ALS (fALS), some also report frontotemporal dementia (FTD), and the other ≈90% are classified as having sporadic ALS (sALS)^8^. To date, over 40 ALS or ALS-FTD causative genes have been reported, the most common being an intronic hexanucleotide repeat expansion (HRE) in *C9orf72* found in 8–12% of ALS patients in Caucasian populations and coding mutations in *SOD1* (encoding superoxide dismutase type 1, SOD1) found in 2–5% of ALS patients^9^. Most of the mutations identified in *SOD1* result in an unstable mutant protein with reduced enzymatic activity^10^. Analysis of SOD1 activity in the blood and SOD1 protein concentration in the cerebrospinal fluid (CSF) of patients were the first biomarkers used for diagnosing ALS and as an endpoint in anti-SOD1 clinical drug trials^10,11^. The rarity of *SOD1* mutations and the finding that seven SOD1 mutants have preserved enzymatic activity limit the clinical use of SOD1 enzymatic analysis, although the finding of reduced SOD1 enzymatic activity may be critical for correctly diagnosing ALS in patients who had been on diagnostic odysseys^10,12,13^. *C9orf72HRE* results in the formation of polydipeptides that can be detected in the CSF, but the results have not yet become part of routine clinical use^14^. Other ALS-causing genes have not resulted in the identification of a specific biomarker for clinical use.

Neurofilaments are neuron-specific cytoskeletal intermediate filament heteropolymers composed of neurofilament light chain (NFL), NF medium chain, and NF heavy chain (NFH) in combination with either α-internexin in the central nervous system or peripherin in the peripheral nervous system^15,16^. Levels of CSF NFL, CSF phosphorylated NFH (pNFH) and serum NFL levels increase with neuronal injury and axonal damage in several neurological disorders, including ALS^17–21^. In longitudinal studies of carriers of *SOD1* and *C9orf72HRE* mutations, NFL levels start to increase 1–12 months prior to the onset of paresis (phenoconversion), continues to rise in early symptomatic patients but then remains relatively stable^22^. NF levels correlate negatively with survival in ALS^17,23–25^. Here, we retrospectively investigated the diagnostic and prognostic value of assaying CSF NFL, CSF pNFH and plasma NFL in samples collected from patients during the diagnostic procedure performed at a specialized university clinic for ALS evaluation and examined whether these biomarkers differed between clinical and genotypic subtypes of ALS.

## Materials and methods

### Participants

All procedures were performed in accordance with the 1964 Declaration of Helsinki and its later amendments. The study was approved by the Medical Ethical Committee (*Forskningsetikkommitten FEK*, nr 1994-135 with later amendments in 1998, 2003, 2014, 2017, 2018) and written informed consent was obtained from all patients to obtain and study the samples and publish the results. The participants were referred to the Department of Neurology, University Hospital of Umeå, Sweden, from 1994 to 2016 to be examined for ALS. The patients were evaluated according to the diagnostic guidelines of the European Federation of Neurological Societies for the clinical management of ALS and diagnosed according to the revised El-Escorial criteria^26,27^. Typically, the diagnostic workup would be performed with the patient in the neurology ward for 3–4 days, and the CSF and plasma samples used in this study were collected at this time. When investigating new patients, since 1994, it has been a custom in our clinic to routinely collect extra blood and CSF samples in case additional material is needed in the diagnostic evaluation after the patient has been discharged from the hospital. These samples are collected and stored with prior written informed consent and may (with approval by the Medical Ethical Committee, FEK) later be used for research purposes should they no longer be needed for further clinical analysis. Patients diagnosed with ALS were followed at regular intervals by a multidisciplinary clinical ALS team. Patients diagnosed with alternative diagnoses were followed by other specialized teams or by local neurologists.

In an initial selection process for inclusion in this study, stored samples from patients referred with a prior medical history of CNS infection, tuberculosis, neuroborreliosis, HIV, syphilis, severe systemic inflammatory disease, severe head trauma or neoplastic conditions or daily medication with anti-inflammatory drugs were excluded. Additionally, patients were excluded if their available CSF and/or plasma samples were insufficient, if they were lost to follow-up (moved abroad or the medical charts were censored for other reasons), participated in a clinical drug trial or their consent forms could not be retrieved.

ALS patient functional status at sampling was assessed retrospectively using the ALS functional rating scale revised (ALSFRS-R)^28^ and the disease progression rate (ΔFS) was calculated as described^29^. Following diagnosis, riluzole was administered to most patients and eventually most patients received a gastrostomy for gastro-enteral feeding and non-invasive ventilation. None of the patients in this study received invasive ventilation through tracheostomy. Survival was defined as the time from onset of paresis to death, usually from respiratory failure due to paresis and/or pneumonia. Disease duration at sampling was defined as the time between onset of paresis and clinical evaluation. Eighteen patients were alive at the time of the study and were excluded from the survival analyses. With separate written informed consent and collection of additional blood for DNA analysis, genetic analyses were performed to evaluate a panel of ALS genes as previously described^30,31^.

For the data analyses, one patient with *C9orf72HRE* who was diagnosed with FTD and a patient with *C9orf72HRE* with FTD and progressive muscular atrophy (PMA) were included in the ALS group. One ALS patient had a *SOD1* G93S mutation and a vesicle-associated membrane protein-associated protein B gene (*VAPB)* mutation and was included in the other *SOD1* mutation group. One ALS patient was heterozygous for both *C9orf72HRE* and *SOD1* D90A and was included in the *C9orf72HRE* mutation group.

### Sample collection and measurements

An extra 2–4 mL of CSF was collected by lumbar puncture during the diagnostic workup, aliquoted and immediately stored at −80 °C. The CSF was not centrifuged before storage. Peripheral blood was collected at the same time by standard venipuncture and collected in EDTA-containing tubes, centrifuged at 1500 × *g* for 15 min, aliquoted and stored at −80 °C. Table 1 summarizes the patient age and disease duration at sampling. The samples included in this study had undergone one freeze–thaw cycle prior to the present analysis. CSF NFL concentrations were analysed with a validated ELISA with intra- and interplate variations of < 8% and < 13%, respectively^32^. CSF NFL analyses were performed in duplicates. Two control samples were not analysed for CSF NFL due to the limited CSF volume. CSF pNFH concentrations were measured with an in-house-developed ELISA with minor modifications with intra- and interplate variations of < 3.9% and < 9.4%, respectively^33^. Regarding CSF pNFH analyses, 226 samples were evaluated in singlicates due to the limited CSF volume. Plasma NFL concentrations were measured using a single-molecule array (SIMOA) assay on an HD-1 Analyzer (Quanterix, Billerica, MA, USA) with intra- and interplate variations of < 10% and < 12%, respectively^34^. Plasma NFL analyses were evaluated in singlicates and in a single batch. Five control samples could not be analysed due to the lack of plasma. CSF NFL, CSF pNFH and plasma NFL analyses were evaluated with the researchers in the laboratory blinded to clinical diagnosis and genotype. The CSF NFL result for one ALS patient was excluded due to a concentration below the calibration curve. For CSF pNFH, biomarker estimates of two ALS patients, one patient from the neuropathies & myelopathies group and one control were excluded because they had concentrations below the calibration curve. For plasma NFL, biomarker estimates of five ALS patients and one patient from the neuropathies & myelopathies group were excluded because they had concentrations below the calibration curve. Patients who had CSF NFL concentrations above the calibration curve (eight ALS patients and one patient in the neuropathies & myelopathies group) were included.

**Table 1:** Age distribution and disease duration at the time of sample collection.

Diagnose group	Age at sampling (years)	Disease duration at sampling (days)
ALS patients total	63.7 ± 13.0	426; 244–680
Spinal onset ALS	60.2 ± 13.1	469; 269–762
Bulbar onset ALS	69.9 ± 11.2	368; 205–506
ALS no known mutation	65.4 ± 13.1	395; 237–624
ALS *SOD1* mutation	54.4 ± 11.9	1144; 437–2007
ALS *C9orf72HRE* mutation	62.7 ± 9.1	358; 194–556
ALS mimics	64.1 ± 12.1	1220; 527–2284

Values are presented as arithmetic mean ± SD or median; Q1–Q3.

### Statistical analysis

Biomarker concentration estimates and patient characteristics were analysed in IBM SPSS Statistics version 26 (International Business Machines Corporation, Armonk, NY, USA). Due to non-normal distributions of estimates, the results are presented as the median and lower and upper quartiles (Q1–Q3) (Table 2). For a prognosis-relevant presentation of biomarkers, ALS patients were also categorized according to the years of survival from symptom onset. The survival times were < 2 years, 2 to < 5 years, 5 to < 10 years and ≥ 10; the biomarker estimates for ALS survival groups are presented as arithmetic mean, standard deviation (SD) and 95% confidence interval (CI) (Table 2). Distributions of biomarker estimates for ALS patients with ≥ 10 years survival are not reported in Table 2 due to the low sample size in this group. Prior to analyses, biomarker estimates were log_10_-transformed to reduce the influence of inhomogeneous variance, reduce distributional skewness, improve normal approximation and decrease the influence of outliers. To compare different relevant groups, one-way analysis of variance (ANOVA) was performed with planned comparison contrast tests and a 95% CI bias-corrected and accelerated bootstrap initially set at 1000 sample runs. In the statistical comparison of CSF NFL concentrations, sample runs had to be increased to 3000 to achieve convergence (stable *p*-values above or below < 0.05). For other statistical tests, there was no need to increase the number of samples per run beyond 1000 to achieve convergence. The Welch test was used when homogeneity of variance could not be assumed. Contrast tests were designed to I) test significant differences between ALS patients and all other groups individually; II) test significant differences between patients with other MNDs compared to those with neuropathies & myelopathies, myopathies and controls; III) test significant differences among spinal onset, bulbar onset and truncal onset ALS patients; IV) test significant differences between ALS patients with mutations in *SOD1*, *C9orf72HRE* and ALS patients with no known mutation and V) test significant differences in the *SOD1* mutation subgroups. The bivariate correlations were investigated using Spearman’s rank-order correlation coefficient (*ρ*). Kaplan–Meier survival analyses were performed for ALS patients whose total survival from symptom onset to death data were available. The time from self-reported symptom onset to death, rather than the time from sampling or diagnosis to death, was used to generate more accurate survival analyses. Kaplan–Meier survival analyses were performed for I) spinal onset and bulbar onset ALS patients and II) ALS patients with mutations in *SOD1*, *C9orf72HRE* and ALS patients with no known mutation. The Mantel–Cox log-rank test was used to test statistical significance for Kaplan–Meier survival analyses. Receiver operating characteristic (ROC) analyses were performed for the original biomarker estimates, and the area under the ROC curve (AUC) was composed for patients with ALS versus ALS mimics for all three biomarkers, where the 95% CI is presented for each AUC. ROC analyses and AUCs for ALS survival are presented for short (< 2 years) and long (≥ 5 years) survival after symptom onset, where patients who were alive during statistical analysis were excluded. AUCs > 0.80 were considered high. Youden’s index highest value (*J*) was used to determine the optimal cut-off for the biomarker concentration, sensitivity, specificity, positive likelihood ratio (LR +) and negative likelihood ratio (LR-). Biomarker ratios were calculated for CSF NFL and CSF pNFH (NFL_CSF_/pNFH_CSF_ ratio) and for plasma NFL and CSF NFL (NFL_plasma_/NFL_CSF_ ratio) using the original biomarker values and are presented as the arithmetic mean ± SD. Statistical differences were considered significant at *p* < 0.05.

**Table 2:** Concentrations of CSF NFL, CSF pNFH and plasma NFL in ALS patients.

	CSF NFL (pg/mL)	CSF pNFH (pg/mL)	Plasma NFL (pg/mL)
**ALS total cohort**	4363; 2813–7135	16,955; 9313–25,707	156; 99–236
Spinal onset ALS	4291; 2639–6994	16,682; 8584–25,397	140; 78–203
Bulbar onset ALS	4885; 3216–7261	18,064; 12,487–26,165	201; 115–291
Truncal onset ALS	3574; 1858–18,596	10,043; 7034–14,253	162; 65–422
ALS no known mutation	4374; 2918–7119	16,446; 9190–25,364	157; 101–238
ALS *SOD1* mutation	3603; 2247–5421	17,598; 8682–28,270	138; 57–180
ALS *SOD1* D90A mutation	2721; 1765–3920	13,318; 6394–21,344	100; 43–157
ALS A4V and other *SOD1* mutations	4769; 3691–5574	33,381; 18,536–43,098	178; 62–354
ALS *C9orf72HRE* mutation	5991; 4119–7652	20,572; 14,331–27,587	181; 125–298
ALS < 2 years survival	7465 ± 5517; 6001–8929	23,206 ± 14,276; 19,271–27,141	291 ± 211; 235–348
ALS 2 to < 5 years survival	5763 ± 4274; 4952–6575	19,617 ± 10,219; 17,620–21,615	188 ± 132; 163–213
ALS 5 to < 10 years survival	4395 ± 4461; 2631–6160	14,528 ± 12,078; 9650–19,407	127 ± 83; 93–160
**ALS mimics**	1205; 758–2234	3651; 2695–5993	43; 25–80
Other MNDs	2260; 1107–5857	8211; 3704–14,485	83; 38–210
Neuropathies & myelopathies	1034; 588–2102	3372; 2201–5131	35; 22–69
Myopathies	992; 840–1150	3056; 2040–5076	40; 22–68
**Controls**	396; 215–892	1516; 1000–2958	12; 8–68

Patients were grouped according to their regions of symptom onset and genotype. ALS mimics are divided into the following groups: other MNDs, neuropathies & myelopathies and myopathies. Biomarker estimates for diagnosis groups are presented as the median; Q1–Q3 and biomarker estimates for ALS survival groups are presented as the arithmetic mean ± SD; 95% CI.

## Results

### Study population

**Figure 1:**
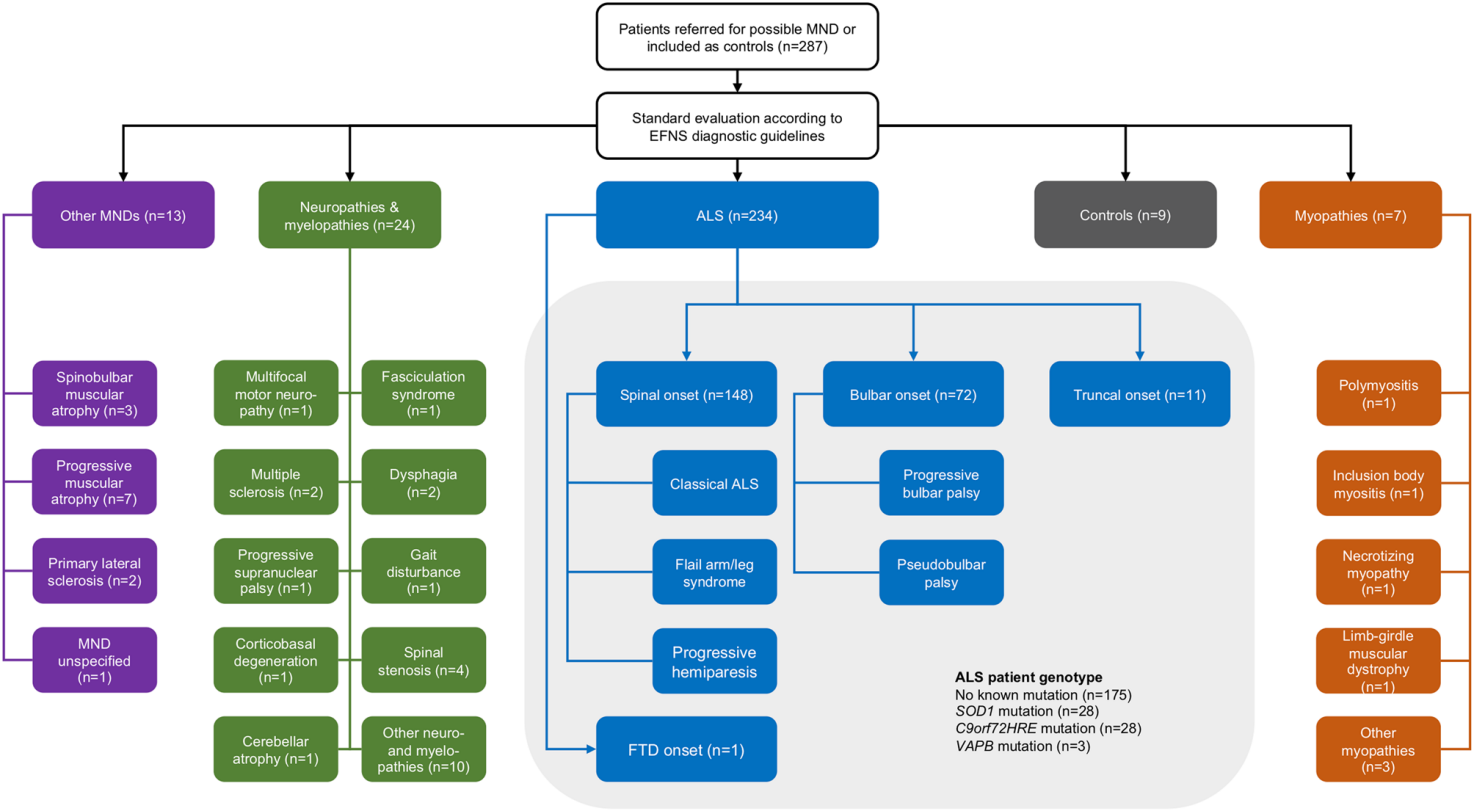
ALS and ALS mimics patient cohort. The study population consisted of patients who underwent investigation for possible motor neuron disease (MND) according to European Federation of Neurological Societies (EFNS) guidelines. The investigation comprised clinical evaluation, neuroimaging, neurophysiological testing (peripheral nerve conduction studies, central motor nerve conduction studies using transcranial motor evoked potentials analysis, and needle electromyography), cerebrospinal fluid and blood analysis. Patients either received an ALS diagnosis (n = 234) or an ALS mimic diagnosis (n = 44). The ALS mimic cohort consisted of patient with other MNDs (n = 13), neuropathies & myelopathies (n = 24) and myopathies (n = 7). A group of controls with no neurological symptoms was recruited for comparison (n = 9). ALS patients were grouped into spinal (n = 148), bulbar (n = 72), truncal (n = 11) or FTD (n = 1) groups according to the site of first onset. ALS patients were also stratified into carriers of mutations in *SOD1* (n = 28), *C9orf72HRE* (n = 28), *VAPB* (n = 3) or patients with no mutation in these genes (n = 175).

The total number of research participants selected for the present study was 287. Participants in the study were categorized as patients with ALS (n = 234) or ALS mimics (n = 44); the latter group consisted of patients with other types of motor neuron diseases (other MNDs) (n = 13), neuropathies & myelopathies (n = 24) or myopathies (n = 7). In addition, controls (n = 9) with no known history of neurological disorders were recruited. The study population is summarized in Fig. 1, and ALS patient characteristics are summarized in Table 3 and Supplementary Table S1. ALS patients were stratified into spinal onset ALS (n = 148), bulbar onset ALS (n = 72), truncal onset ALS (n = 11) and FTD onset ALS (n = 1). ALS patients were also stratified into those with mutations in *SOD1* (n = 28), those with *C9orf72HRE* mutations (n = 28), those with *VAPB* mutations (n = 3) or other ALS patients without mutations in these genes (n = 175). Patients with *SOD1* mutations were further stratified into *SOD1* D90A homozygous (n = 14), D90A heterozygous (n = 3), A4V heterozygous (n = 2) and other *SOD1* mutation (n = 9) groups.

**Table 3:** ALS patient characteristics.

**ALS patient characteristics**	
ALS symptom onset: spinal onset/bulbar onset/truncal onset/FTD onset/unknown	148 (63.2)/72 (30.8)/11 (4.7)/1 (0.4)/2 (0.9)
Sex: male/female	124 (53.0)/110 (47.0)
Mutation status: No known mutation/*SOD1*/*C9orf72HRE*/*VAPB*	175 (74.8)/28 (12.0)/28 (12.0)/3 (1.2)
ALSFRS-R at sampling	42; 38–45
ΔFS at sampling	0.45; 0.23–0.90
Age of symptom onset: all ALS/spinal onset ALS/bulbar onset ALS	61.7 ± 13.4/57.9 ± 13.3/68.7 ± 11.4
Age of symptom onset: No known mutation/*SOD1*/*C9orf72HRE*	64.0 ± 13.2/50.3 ± 11.7/61.1 ± 9.0
Survival after symptom onset: all ALS/spinal onset ALS/bulbar onset ALS	2.9; 1.8–4.4/3.3; 2.3–5.4/2.5; 1.5–3.1
Survival after symptom onset: No known mutation/*SOD1*/*C9orf72HRE*	2.8; 1.7–4.1/5.2; 2.8–14.1/2.7; 1.8–3.3
Age at death: all ALS/spinal onset ALS/bulbar onset ALS	67.1 ± 11.9/64.3 ± 11.9/71.7 ± 10.9
Age at death: No known mutation/*SOD1*/*C9orf72HRE*	68.4 ± 11.7/59.0 ± 13.1/65.1 ± 8.8
Survival groups: < 2 years/2 to < 5 years/5 to < 10 years/ ≥ 10 years	57 (26.8)/110 (51.6)/27 (12.7)/19 (8.9)

Data are presented as either frequency and percentage, arithmetic mean ± SD or median; Q1–Q3. Age of symptom onset, survival after symptom onset and age at death are presented in years.

### CSF NFL and CSF pNFH

**Figure 2:**
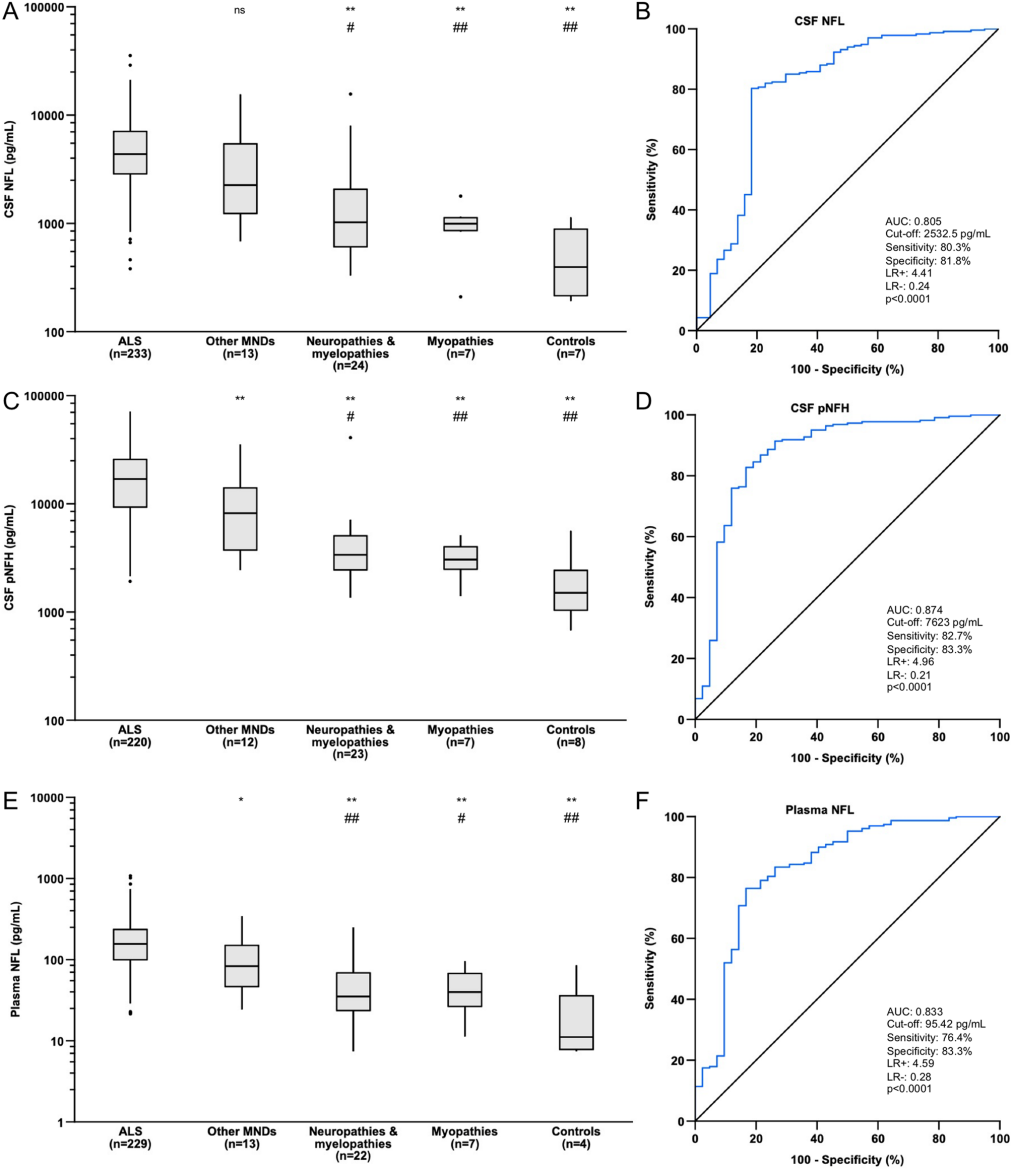
Neurofilament levels and ROC analyses. **(A)** Boxplots of CSF NFL concentration estimates of participants in the ALS, ALS mimics and control groups. **(B)** ROC analysis of participants in the ALS versus ALS mimics groups for CSF NFL. **(C)** Boxplots of CSF pNFH concentration estimates in participants in the ALS, ALS mimics and control groups. **(D)** ROC analysis of participants in the ALS versus ALS mimics groups for CSF pNFH. **(E)** Boxplots of plasma NFL concentration estimates of participants in the ALS, ALS mimics and control groups. **(F)** ROC analysis of participants in the ALS versus ALS mimics groups for plasma NFL. ALS patients versus participants with other MNDs, participants with neuropathies & myelopathies, participants with myopathies and controls: *p < 0.05, **p < 0.01, *ns*  non-significant. Participants with other MNDs versus participants with neuropathies & myelopathies, participants with myopathies and controls: #*p* < 0.05, ##*p* < 0.01.

**Figure 3:**
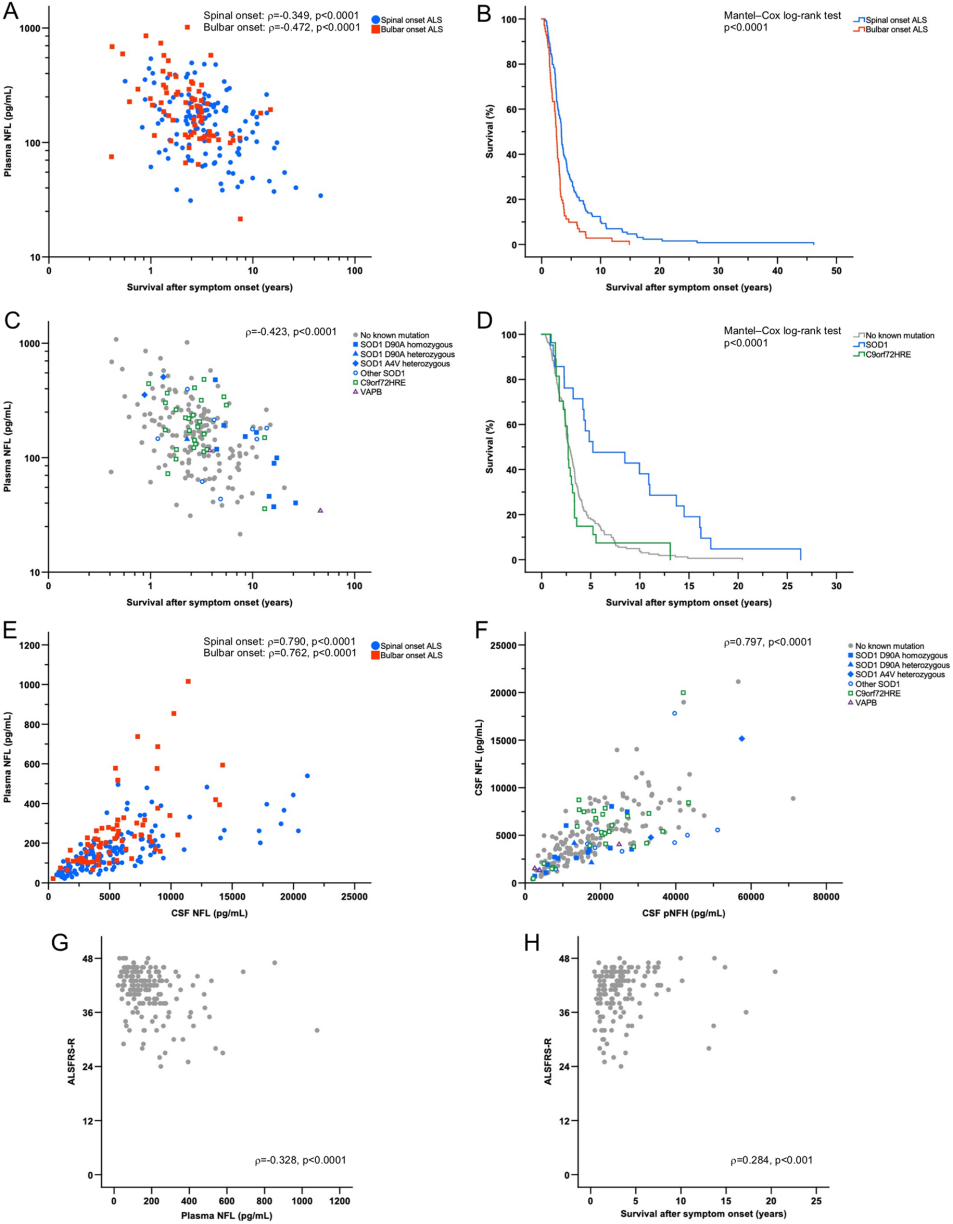
Biomarker correlation to survival, survival analyses in clinical symptom onset and genotype groups, biomarker ratios and ALSFRS-R in ALS patients. **(A)** Correlation between plasma NFL and survival after symptom onset stratified by spinal and bulbar symptom onset. **(B)** Kaplan–Meier survival analysis for spinal onset versus bulbar onset ALS patients. **(C)** Correlation between plasma NFL and survival after symptom onset for participants in the total ALS cohort. **(D)** Kaplan–Meier survival analysis between patients with no known mutation, *SOD1* mutation and *C9orf72HRE* mutation (*p*-value presented for overall comparisons). **(E)** Ratios and correlations between plasma NFL and CSF NFL stratified for spinal onset and bulbar onset ALS patients. **(F)** Correlation between CSF NFL and CSF pNFH for participants in the total ALS cohort. **(G)** Correlation between ALSFRS-R and plasma NFL in ALS patients. **(H)** Correlation between ALSFRS-R and survival after symptom onset in ALS patients.

Concentrations of CSF NFL and CSF pNFH are presented in Figs. 2A,C and 3F and Table 2. ALS patients had significantly higher CSF NFL and CSF pNFH levels than patients with neuropathies & myelopathies (*p* < 0.01 for both), patients with myopathies (*p* < 0.01 for both) and controls with no neurological disorder (*p* < 0.01 for both) (Fig. 2A,C). There was a statistically significant difference when comparing CSF pNFH in ALS patients to that of patients with other MNDs (*p* < 0.01) (Fig. 2C) but not when comparing CSF NFL (*p* > 0.05) (Fig. 2A). Patients in the other MNDs group had significantly higher CSF NFL and CSF pNFH levels than patients with neuropathies & myelopathies (*p* < 0.05 for both), myopathies (*p* < 0.01 for both) and controls with no neurological disorder (*p* < 0.01 for both) (Fig. 2A,C). Neither CSF NFL levels nor CSF pNFH levels were significantly different among ALS patients with spinal onset, bulbar onset or truncal onset (*p* > 0.05 for overall ANOVA).

In patients in the ALS group, the concentrations of CSF NFL and CSF pNFH in patients with no known mutation, *SOD1* mutation and *C9orf72HRE* mutation did not show any statistically significant difference (*p* > 0.05 for overall ANOVA). When stratifying ALS patients into *SOD1* mutation subgroups, ALS patients with *SOD1* D90A homozygous or heterozygous mutations combined showed significantly lower levels of CSF pNFH compared to those with *SOD1* A4V heterozygous and other ALS *SOD1* mutations combined (CSF pNFH; *p* < 0.01, CSF NFL; *p* > 0.05). In ALS patients, the concentrations of CSF NFL and CSF pNFH were significantly correlated (*ρ* = 0.797, *p* < 0.0001) (Fig. 3F). ALSFRS-R was negatively correlated with CSF NFL levels (*ρ* = −0.241, *p* < 0.01) but not with CSF pNFH levels (p > 0.05). Furthermore, ΔFS was correlated with CSF NFL levels (*ρ* = 0.355, *p* < 0.0001) and CSF pNFH levels (*ρ* = 0.346, *p* < 0.0001). There was no significant correlation between freezer storage time and the concentrations of CSF NFL or between freezer storage time and CSF pNFH in the patients in the ALS or ALS mimics groups (*p* > 0.05 for both).

### Plasma NFL

Concentrations of plasma NFL are presented in Figs. 2E, and 3A,C,E,G and Table 2. ALS patients had significantly higher plasma NFL concentrations than patients with other MNDs (*p* < 0.05), patients with neuropathies & myelopathies (*p* < 0.01), patients with myopathies (*p* < 0.01) and controls with no neurological disorders (*p* < 0.01) (Fig. 2E). Patients with other MNDs had significantly higher plasma NFL levels than patients with neuropathies and myelopathies (*p* < 0.01), patients with myopathies (*p* < 0.05) and controls with no neurological disorder (*p* < 0.01) (Fig. 2E).

Patients with bulbar onset ALS had significantly higher plasma NFL concentrations than patients with spinal onset ALS (*p* < 0.01). There was no statistically significant difference when comparing the levels of plasma NFL of ALS patients with truncal onset to those of ALS patients with spinal onset or bulbar onset (*p* > 0.05 for both). In ALS patients, the concentrations of plasma NFL were significantly correlated with CSF NFL (*ρ* = 0.773, *p* < 0.0001) and CSF pNFH (*ρ* = 0.696, *p* < 0.0001). When stratified by the region of symptom onset, patients with spinal onset or bulbar onset ALS both showed a higher correlation between plasma NFL and CSF NFL levels (*ρ* = 0.790, *p* < 0.0001 and *ρ* = 0.762, *p* < 0.0001, respectively) (Fig. 3E) than all ALS patients as a whole group. The plasma NFL concentration was significantly higher in ALS patients with *C9orf72HRE* mutations than in patients with *SOD1* mutations (*p* < 0.05). No significant difference in plasma NFL concentration was found by comparing ALS patients with no known mutations to ALS patients with *SOD1* or *C9orf72HRE* mutations (p > 0.05 for both). When stratifying ALS patients into spinal and bulbar onset groups, statistically significant differences in plasma NFL concentrations in spinal onset ALS patients were observed when comparing patients with *C9orf72HRE* mutations to both patients who were *SOD1* mutation carriers (*p* < 0.01) and ALS patients with no known mutations (*p* < 0.05); however, there was no statistically significant difference among different genotype groups in bulbar onset ALS patients (*p* > 0.05 for overall ANOVA). ALS patients homozygous or heterozygous mutation for the D90A *SOD1* mutation combined showed significantly lower plasma NFL concentrations compared to patients with *SOD1* A4V and other *SOD1* mutations combined (*p* < 0.01). Plasma NFL concentration was negatively correlated with ALSFRS-R (*ρ* = −0.328, *p* < 0.0001) (Fig. 3G) and positively correlated with ΔFS (*ρ* = 0.426, *p* < 0.0001). There was no statistically significant correlation between plasma NFL concentrations and freezer storage time from samples collected from ALS patients and ALS mimics (*p* > 0.05 for both).

### ROC and survival analysis

ROC analyses showed high AUCs when ALS patients were compared with ALS mimics (Fig. 2B,D,F). CSF pNFH had the highest AUC (AUC: 0.874; 0.803–0.944) (*J*: 7623 pg/mL, sensitivity: 82.7%, specificity: 83.3%, LR + : 4.96, LR−: 0.21, *p* < 0.0001) (Fig. 2D), followed by plasma NFL concentration (AUC: 0.833; 0.756–0.910) (*J*: 95.42 pg/mL, sensitivity: 76.4%, specificity: 83.3%, LR + : 4.59, LR−: 0.28, *p* < 0.0001) (Fig. 2F) and CSF NFL (AUC: 0.805; 0.719–0.891) (*J*: 2532.5 pg/mL, sensitivity: 80.3%, specificity: 81.8%, LR + : 4.41, LR−: 0.24, *p* < 0.0001) (Fig. 2B) when comparing patients with ALS to ALS mimics . Survival after ALS symptom onset was significantly negatively correlated with CSF NFL (*ρ* = −0.360, *p* < 0.0001), CSF pNFH (*ρ* = −0.295, *p* < 0.0001) and plasma NFL levels (*ρ* = −0.423, *p* < 0.0001). Furthermore, disease duration at sampling was significantly positively correlated with total survival in ALS patients (*ρ* = 0.600, *p* < 0.0001). There were significant differences in survival between spinal onset ALS and bulbar onset ALS patients (*p* < 0.0001) (Fig. 3B) and among ALS patient genotype groups (*p* < 0.0001 for overall comparisons, *p* < 0.01 for ALS patients with *SOD1* mutations versus *C9orf72HRE* mutations) (Fig. 3D). Due to significant differences in plasma NFL concentrations and survival between spinal onset and bulbar onset ALS patients, a scatterplot revealed a significant negative correlation between plasma NFL levels and survival for patients with spinal onset ALS (*ρ* = −0.349, *p* < 0.0001) and bulbar onset ALS (*ρ* = −0.472, *p* < 0.0001) (Fig. 3A). ROC analyses of ALS patients with short and long survival times showed the highest AUC for plasma NFL levels (AUC: 0.800; 0.716–0.884) (*J*: 205.66 pg/mL, sensitivity: 58.9%, specificity: 91.1%, LR + : 6.63, LR−: 0.45) (*p* < 0.0001), followed by CSF NFL levels (AUC: 0.751; 0.655–0.846) (*J*: 4265 pg/mL, sensitivity: 70.2%, specificity: 73.9%, LR + : 2.69, LR−: 0.40) (*p* < 0.0001) and CSF pNFH (AUC: 0.721; 0.618–0.825) (*J*: 18,537 pg/mL, sensitivity: 56.6%, specificity: 79.1%, LR + : 2.70, LR−: 0.55) (*p* < 0.0001). ALSFRS-R was significantly positively correlated with survival (*ρ* = 0.284, *p* < 0.001) (Fig. 3H) and significantly negatively correlated with disease duration at sampling in ALS patients (*ρ* = −0.180, *p* < 0.05). Furthermore, ΔFS was significantly negatively correlated with survival in ALS patients (*ρ* = −0.659, *p* < 0.0001).

### NFL and pNFH ratios

There was no significant difference in the NFL_CSF_/pNFH_CSF_ ratio among ALS patients, ALS mimic groups and controls (*p* > 0.05 for overall ANOVA) or between ALS symptom onset groups (*p* > 0.05 for overall ANOVA). Bulbar onset ALS patients had a significantly higher NFL_plasma_/NFL_CSF_ ratio (0.046 ± 0.021) than spinal onset ALS patients (0.034 ± 0.016) (*p* < 0.01) (Fig. 3E). Although patients with *C9orf72HRE* mutations had notably higher NFL_CSF_/pNFH_CSF_ ratios (0.302 ± 0.129) than patients with *SOD1* mutations (0.242 ± 0.111) (Fig. 3F), this difference did not reach statistical significance (*p* > 0.05 for overall ANOVA).

## Discussion

Making a correct ALS diagnosis early after symptom onset has become even more important since observations from clinical drug trials show that patients enrolled early after onset of first paresis frequently have better outcomes than patients enrolled later^35^. The launch of promising bespoken therapy trials targeting *SOD1*, *C9orf72HRE* and fused in sarcoma gene (*FUS*) in symptomatic patients with mutations in these genes further emphasizes the need to be able to diagnose ALS early in the disease course. Additionally, experimental personalized gene therapy in adult asymptomatic carriers of these mutations will begin soon. A major obstacle is determining when to initiate prophylactic personalized medicine; the wide range in age at onset of the first symptom, heterogeneity in the first clinical presentation and the frequent occurrence of reduced disease penetrance in families with mutations pose a challenge^10^. Reliable biomarkers showing that the neurodegenerative process has begun or not begun are urgently needed. Although there is emerging evidence that in patients with a *SOD1* mutation, the mutant SOD1 protein forms cytotoxic prion-like species that propagate through the motor system^36^, no research group has yet successfully been able to detect such SOD1 prions in in vivo material from ALS patients. Presently, we must use less informative downstream biomarkers for diagnosing early ALS and for differentially diagnosing ALS.

In accordance with reports in smaller cohorts^21,37^, the present study confirms that ALS patients have significantly higher CSF and plasma levels of NFs than patients with a number of relevant ALS mimics. In this larger study, the overlap between the groups was small, demonstrating the usefulness of assaying NFs in the differential diagnosis of ALS. In particular, the levels of CSF pNFH and plasma NFL were significantly higher in patients in the ALS group than in patients in the other MNDs groups. When discriminating ALS from ALS mimics, CSF pNFH had a higher AUC than CSF NFL and plasma NFL levels, suggesting that CSF pNFH is a better biomarker assay in differentiating ALS from clinically relevant mimics in this study.

It has previously been reported that neurofilament levels in the CSF do not distinguish ALS patients according to the site of ALS symptom onset^38^. The present study shows similar results in CSF, although CSF NFL and CSF pNFH levels were notably (non-significantly) higher in bulbar onset ALS patients than in spinal onset ALS patients. In a previous study, bulbar onset ALS patients had close to significantly higher plasma pNFH levels than spinal onset ALS patients but without a statistically significant difference in CSF^39^. In the present larger study, we found that plasma NFL levels were significantly higher in bulbar onset ALS patients than in spinal onset ALS patients. Furthermore, bulbar onset ALS patients showed a significantly higher NFL_plasma_/NFL_CSF_ ratio than spinal onset ALS patients.

Bulbar onset ALS is associated with a worse prognosis than spinal onset ALS^3^, and an earlier study suggested that NFL concentrations are related to the volume of damaged neuronal tissue^33^. The usefulness of NFL and pNFH levels in the prognostication of ALS is controversial^24,40,41^. Here, we found that ALS patients with lower levels of all three biomarkers survived longer than ALS patients with higher levels. Speculatively, the lower levels of neurofilaments and longer survival in some patients suggest that axonal loss progresses slower in these patients; thus, fewer neurofilaments are displaced into the CFS and plasma. Hence, interventions that markedly lower neurofilament levels in the CSF and plasma slow the progression of neuronal loss. The present finding that the plasma NFL concentration is higher in bulbar onset ALS patients than in spinal onset ALS patients is therefore in accordance with the worse prognosis in bulbar onset ALS patients than in spinal onset ALS patients^3^.

Hypermetabolism and weight loss correlate with worse prognosis in ALS^42–44^. Weight loss relates to the site of symptom onset and dysphagia in ALS, and survival has been shown to be affected by weight loss in both spinal onset and bulbar onset ALS patients^45^. However, the effect of weight loss and dysphagia on survival did not differ significantly between spinal or bulbar onset ALS patients^45^, which might suggest worse survival in bulbar onset patients even if the same relative degree of weight loss was present in both groups. Thus, the significantly higher plasma NFL levels in bulbar onset ALS patients might suggest that the worse prognosis is primarily due to a more aggressive neurodegenerative process. In epidemiological studies, the incidence of bulbar onset ALS is higher in women and in patients as age increases^46^. This is also the case in our study cohort, making the findings of differences in plasma NFL concentration between spinal and bulbar onset ALS patients clinically applicable.

In this study, a subset of ALS patients were carriers of mutations in *SOD1*, *C9orf72* or *VAPB*. ALS patients carrying *C9orf72HRE* mutations have significantly higher CSF pNFH and serum pNFH levels than patients without a mutation in *C9orf72*^47,48^. Additionally, *SOD1* mutation carriers had significantly lower CSF NFL levels than patients with no *SOD1* mutations^17^. Our new results support these results, showing higher plasma NFL levels and worse survival in ALS patients with *C9orf72HRE* mutations than in patients with *SOD1* mutations. Stratifying ALS patients into spinal and bulbar symptom onset, patients in the spinal onset group with a *C9orf72HRE* had significantly higher plasma NFL levels than ALS patients with a *SOD1* mutation. This finding further supports the importance of evaluating neurodegeneration in ALS patients with regard to both clinical symptoms at onset and genotype.

Since patients with primary lateral sclerosis (PLS) with only upper motor neuron engagement or PMA with only lower motor neuron engagement generally have a better prognosis than ALS patients^49,50^, it would be unfortunate to enrol patients with PLS or PMA in an ALS intervention trial. It is therefore important to clearly differentiate between early ALS and early PLS or PMA diseases. A study found that ALS patients have significantly higher serum NFL concentrations than PLS patients^37^. We therefore propose further studies also using upper motor neuron-specific biomarkers (e.g., α-internexin) and lower motor neuron-specific biomarkers (e.g., peripherin).

Limitations of the present study include retrospective assessment of ALSFRS-R (the scale did not exist when the first patients were seen in our clinic), some of the CSF pNFH analyses were performed in singlicates and plasma pNFH analyses were not performed. The samples included in the present study were collected over several years and had undergone one freeze–thaw cycle prior to analysis. Arguably, the concentrations of NFL and pNFH could potentially be confounded by these factors. Reportedly, both NFL and NFH can withstand four freeze–thaw cycles without affecting concentrations significantly, and the Arrhenius plot for NFH suggests stable properties for storage at −80 °C for an extended time^51^. This stability may be due to the phosphorylated carboxy-terminal of pNFH^52,53^. In our dataset, there was no significant correlation between freezer storage time and concentrations of all three biomarkers, indicating that the samples were not significantly degraded due to storage time and therefore were comparable.

The strengths of the study are the analyses of NFL and pNFH levels in CSF and NFL in plasma samples from a large group of clinically relevant and commonly encountered ALS patients and ALS mimics; all performed in the same laboratory and blinded to clinical data. Moreover, the study extensively characterized patient cohorts with clinical and genotype information from patients from the same site. The long follow-up observation time following the collection of the analysed samples is of importance as it provides enough time for a correct diagnosis of slowly progressing ALS. Finally, 26 of the ALS patients later underwent post-mortem autopsy confirming the diagnosis.

In conclusion, our study results confirm earlier findings on neurofilament and ALS^6,18,19,24,25,37,38,41,54–58^ but add new knowledge on the comparative performance of plasma and CSF neurofilaments in a clinical context, thus emphasizing the importance of a correct ALS diagnosis early and prognostication. Regarding the diagnostic properties, all three biomarkers are of clinical value in affirming an ALS diagnosis and excluding potential ALS mimics. CSF pNFH showed the highest AUC in terms of differentiating ALS from ALS mimics. Plasma NFL analysis has the advantage that it does not require a lumbar puncture, has only a minimal difference in diagnostic performance compared to CSF NFL levels and shows the highest AUC in terms of prognosticating ALS short and long survival.

## Supplementary Information

Supplementary Table S1.

## Data Availability

Data used and analysed in the present study will be available from the corresponding author upon reasonable request from other investigators adhering to the European Union General Data Protection Regulation (EU) 2016/679 (GDPR).
