# Supplementary material for: Neurofilaments can differentiate ALS subgroups and ALS from common diagnostic mimics
Source: Sci Rep. 2021 Nov 11;11:22128. doi: 10.1038/s41598-021-01499-6 (PMC8585882; doi:10.1038/s41598-021-01499-6)
Supplement: Supplementary file 1 — Supplementary Table S1. [file 41598_2021_1499_MOESM1_ESM.pdf]

Neurofilaments can differentiate ALS subgroups and ALS from common diagnostic mimics.

Authors: Arvin Behzadi, Fani Pujol-Calderón, Anton E. Tjust, Anna Wuolikainen, Kina Höglund, Karin Forsberg, Erik Portelius, Kaj Blennow, Henrik Zetterberg, Peter Munch Andersen

Supplementary Table S1. Patient demographics.

**ALS patient characteristics**	
ALS symptom onset: spinal onset/bulbar onset/truncal onset/FTD onset/unknown	148 (63.2)/72 (30.8)/11 (4.7)/1 (0.4)/2 (0.9)
Sex: male/female	124 (53.0)/110 (47.0)
Sex spinal onset: male/female	86 (58.1)/62 (41.9)
Sex bulbar onset: male/female	30 (41.7)/42 (58.3)
Mutation status: no known mutation/*SOD1*/*C9orf72HRE*/*VAPB*	175 (74.8)/28 (12.0)/28 (12.0)/3 (1.2)
ALSFRS-R at sampling: all ALS/spinal onset ALS/bulbar onset ALS	42; 38-45/42; 38-45/43; 39-45
ALSFRS-R at sampling: no known mutation/*SOD1*/*C9orf72HRE*	42; 39-45/41; 36-44/41; 38-45
ΔFS at sampling: all ALS/spinal onset ALS/bulbar onset ALS	0.45; 0.23-0.90/0.38; 0.22-0.81/0.64; 0.27-1.08
ΔFS at sampling: no known mutation/*SOD1*/*C9orf72HRE*	0.40; 0.23-0.89/0.34; 0.08-1.00/0.52; 0.38-0.92
Age at symptom onset: all ALS/spinal onset ALS/bulbar onset ALS	61.7 ± 13.4/57.9 ± 13.3/68.7 ± 11.4
Age at symptom onset: no known mutation/*SOD1*/*C9orf72HRE*	64.0 ± 13.2/50.3 ± 11.7/61.1 ± 9.0
Survival after symptom onset: all ALS/spinal onset ALS/bulbar onset ALS	2.9; 1.8-4.4/3.3; 2.3-5.4/2.5; 1.5-3.1
Survival after symptom onset: no known mutation/*SOD1*/*C9orf72HRE*	2.8; 1.7-4.1/5.2; 2.8-14.1/2.7; 1.8-3.3
Age at death: all ALS/spinal onset ALS/bulbar onset ALS	67.1 ± 11.9/64.3 ± 11.9/71.7 ± 10.9
Age at death: no known mutation/*SOD1*/*C9orf72HRE*	68.4 ± 11.7/59.0 ± 13.1/65.1 ± 8.8
Survival groups: <2 years/2 to <5 years/5 to <10 years/≥10 years	57 (26.8)/110 (51.6)/27 (12.7)/19 (8.9)
**ALS mimics characteristics**	
Sex: male/female	31 (70.5)/13 (29.5)
Age of symptom onset	57.5 ± 14.2
Survival after symptom onset	5.9; 2.5-14.7
Age at death	73.2 ± 13.8

Data are presented as either frequency and percentage, arithmetic mean ± SD or median; Q1-Q3. Age of symptom onset, survival after symptom onset and age at death are presented in years. ALS: amyotrophic lateral sclerosis, FTD: Fronto-temporal dementia, *SOD1*: superoxide dismutase type 1 gene, *C9orf72HRE*: hexanucleotide repeat expansion in the *C9orf72* gene, *VAPB*: Vesicle-Associated Protein type B gene, ALSFRS-R: revised ALS functional rating scale, ΔFS: disease progression rate (from onset of first paresis to sampling event).
